# Towards prognostic functional brain biomarkers for cervical myelopathy: A resting-state fMRI study

**DOI:** 10.1038/s41598-019-46859-5

**Published:** 2019-07-18

**Authors:** Shota Takenaka, Shigeyuki Kan, Ben Seymour, Takahiro Makino, Yusuke Sakai, Junichi Kushioka, Hisashi Tanaka, Yoshiyuki Watanabe, Masahiko Shibata, Hideki Yoshikawa, Takashi Kaito

**Affiliations:** 1 Orthopaedic Surgery, Osaka University Graduate School of Medicine, Suita, Osaka Japan; 2 Anesthesiology and Intensive Care Medicine, Osaka University Graduate School of Medicine, Suita, Osaka Japan; 3 Center for Information and Neural Networks, National Institute of Information and Communications Technology, Suita, Osaka Japan; 4 Immunology Frontiers Research Center, Osaka University, Suita, Osaka Japan; 5 Computational and Biological Learning Laboratory, Department of Engineering, University of Cambridge, Cambridge, UK; 6 Diagnostic and Interventional Radiology, Osaka University Graduate School of Medicine, Suita, Osaka Japan; 7 Faculty of Health Sciences, Naragakuen University, Nara, Japan

**Keywords:** Prognostic markers, Spinal cord diseases

## Abstract

Recently, there has been increasing interest in strategies to predict neurological recovery in cervical myelopathy (CM) based on clinical images of the cervical spine. In this study, we aimed to explore potential preoperative brain biomarkers that can predict postoperative neurological recovery in CM patients by using resting-state functional magnetic resonance imaging (rs-fMRI) and functional connectivity (FC) analysis. Twenty-eight patients with CM and 28 age- and sex-matched healthy controls (HCs) underwent rs-fMRI (twice for CM patients, before and six months after surgery). A seed-to-voxel analysis was performed, and the following three statistical analyses were conducted: (i) FC comparisons between preoperative CM and HC; (ii) correlation analysis between preoperative FCs and clinical scores; and (iii) postoperative FC changes in CM. Our analyses identified three FCs between the visual cortex and the right superior frontal gyrus based on the conjunction of the first two analyses [(i) and (ii)]. These FCs may act as potential biomarkers for postoperative gain in the 10-second test and might be sufficient to provide a prediction formula for potential recovery. Our findings provide preliminary evidence supporting the possibility of novel predictive measures for neurological recovery in CM using rs-fMRI.

## Introduction

Cervical myelopathy (CM) is a common disorder characterized by a constellation of symptoms and signs caused by a disruption of neural transmission at the cervical spinal cord. The usefulness of several neuroimaging techniques targeting the cervical spine to predict neurological recovery in CM has been investigated. Currently, magnetic resonance imaging (MRI) is regarded as a gold standard for diagnostic and predictive imaging^1,2^. However, it remains controversial whether conventional MRI of the cervical spine (e.g., high signal intensity on T2-weighted MRI) is a useful predictive imaging biomarker^3–7^ because of the limited information obtained from this anatomically small area, and the involvement of both static and dynamic cord compression in spinal cord damage^8^. Therefore, simple, accurate, and non-invasive imaging biomarkers are needed to predict neurological recovery in CM.

Recently, resting-state functional MRI (rs-fMRI), which enables visualisation of brain functional connectivity (FC) in the absence of tasks, has attracted attention in various fields, including neurodegenerative diseases^9,10^ and chronic pain^11,12^. FC refers to the functionally integrated relationship between spatially separated brain regions. Rs-fMRI is particularly suitable for use with CM patients because of their difficulties in performing tasks due to paralysis^13–15^.

Previous studies exploring the use of MRI in CM have analysed only preoperative FCs or FCs of a specific anatomical region (e.g., sensorimotor cortex)^16,17^. Although a previous study investigated preoperative visual cortex neural activity and FC alterations in cervical spondylotic myelopathy (CSM) patients^18^, no studies have investigated postoperative changes in FCs across whole brain networks or the correlation between FC changes and clinical recovery. If such a correlation is established, then it might lead to a useful biomarker for decision-making of surgical indication.

In this study, we aimed to explore preoperative brain biomarkers that can predict postoperative neurologic recovery in CM patients. We conducted three analyses, as follows: (i) comparison of FC in preoperative CM and healthy controls (HC); (ii) correlation analysis between preoperative FCs and clinical recovery in CM; and (iii) postoperative FC changes in CM.

## Results

### Participant characteristics

Table 1 presents the demographics of the CM and HC groups. In the CM group, clinical outcomes including scores for the 10-second test^19^, the Japanese Orthopaedic Association (JOA) score^20^, and Japanese Orthopaedic Association Cervical Myelopathy Evaluation Questionnaire (JOACMEQ) score^21^ showed significant postoperative improvements (Table 2).

**Table 1:** Participant characteristics.

Variable	CM	HC
Participants (n)	28	28
Sex (m/f)	14/14	14/14
Age, years	66.5 ± 10.9 (44–79)	66.5 ± 11.0 (43–79)
Handedness (right/left)	26/2	26/2
Medication		
NSAIDs	2	0
Pregabalin	3	0
Tramadol/acetaminophen	1	0
Sedative-hypnotics	7	0
Change in the medication	1 (Stop of pregabalin)	NA
Disease duration (month)	36.4 ± 55.1 (3–246)	NA

CM, cervical myelopathy; HC, healthy control; m, male; f, female; NSAIDs, nonsteroidal anti-inflammatory agents; NA, not applicable.

Data are presented as mean ± SD (range) for continuous variables.

**Table 2:** Patient clinical score.

Variable	Preop	Postop	p
JOA scores			
Upper-extremity motor score	2.2 ± 1.0 (−0.5–4)	3.4 ± 0.6 (2.5–4)	<0.001
Lower-extremity motor score	2.3 ± 0.9 (0.5–4)	2.9 ± 1.0 (1–4)	0.004
Upper-extremity sensory score	1.0 ± 0.3 (0.5–1.5)	1.3 ± 0.4 (1–2)	0.001
Trunk sensory score	1.7 ± 0.5 (1–2)	2.0 ± 0.1 (1.5–2)	0.004
Lower-extremity motor score	1.2 ± 0.5 (0.5–2)	1.7 ± 0.4 (1–2)	<0.001
Sphincter score	2.4 ± 0.7 (1–3)	2.8 ± 0.5 (1–3)	0.009
Total	10.8 ± 2.6 (4–15)	14.0 ± 2.0 (9–17)	<0.001
JOACMEQ scores			
Neck function	62.0 ± 27.4 (0–100)	77.9 ± 27.6 (10–100)	0.001
Upper extremity function	71.1 ± 25.7 (5–100)	87.6 ± 15.7 (42–100)	0.028
Lower extremity function	55.3 ± 29.8 (0–100)	73.5 ± 23.1 (36–100)	0.008
Bladder function	70.4 ± 19.0 (38–100)	79.7 ± 17.9 (38–100)	<0.001
QOL	41.9 ± 24.0 (2–94)	53.4 ± 21.5 (16–97)	0.023
10-second test (lower side)*	15.6 ± 3.5 (11–21)	27.4 ± 7.8 (12–41)	0.004
Operative gain in 10-second test	NA	11.8 ± 6.6 (0–21)	
VAS			
Pain or numbness in upper extremity (mm)	55.5 ± 28.0 (8–100)	45.9 ± 31.3 (0–100)	0.246

JOA, Japanese Orthopaedic Association; Japanese Orthopaedic Association Cervical Myelopathy Evaluation Questionnaire; QOL, quality of life; NA, not applicable; VAS, visual analogue scale.

Data are presented as mean ± SD (range) for continuous variables.

*The number for the 10-second test was represented by that on the lower side.

### Comparison of the FCs between the CM and HC groups

There were 25 clusters showing significantly decreased FC in the preoperative CM group compared with the HC group (Table S1). Furthermore, there were 5 clusters showing significantly increased FC in this group (Table S2).

### Correlation between preoperative FCs and clinical scores in the CM group

We next investigated correlations between preoperative FCs and clinical score changes in the CM group. Because scores related to lower extremity function can be affected by joint disorders such as knee osteoarthritis^22^, we applied only scores related to upper extremity function, these being the 10-second test, JOA upper-extremity motor score (JOA-UEM), JOA upper-extremity sensory score (JOA-UES), and JOACMEQ upper-extremity function score (JOACMEQ-UEF), for this analysis.

**Figure 1:**
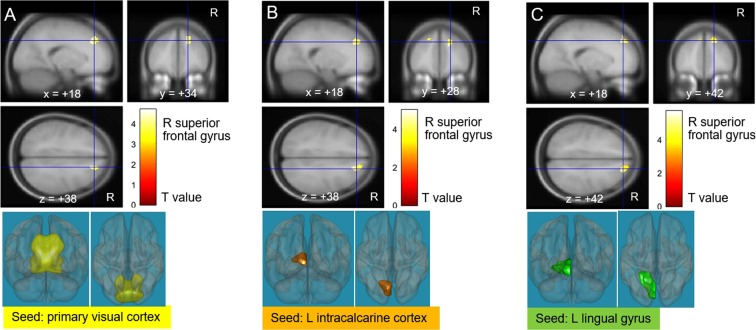
Correlation analysis between preoperative functional connectivities (FCs) and the 10-second test recovery in cervical myelopathy (CM) patients. After using the mask with CM < healthy control (HC), three visual areas (the primary visual cortex, left intracalcarine cortex, and left lingual gyrus) displayed a positive association between the 10-second test and FC with the right superior frontal gyrus. R, right; L, left.

There were 15 clusters showing a significantly positive correlation between FCs and postoperative gain in the 10-second test (Table S3). Subsequently, we analysed whether there is any overlap of clusters showing a significant difference in FC between preoperative CM and HC with those showing a significant correlation between FC and postoperative gain in the 10-second test. Three clusters (FCs of the primary visual cortex, left intracalcarine cortex, and left lingual gyrus with the right superior frontal gyrus) overlapped with clusters showing a significant FC difference between CM and HC (Fig. 1). In contrast, one cluster showing a significant negative correlation between FCs and postoperative gain in the 10-second test did not overlap with clusters with a significant FC difference between CM and HC (Table S4). Similarly, clusters showing significantly positive or negative correlations with the JOA-UEM (Tables S5 and S6), JOA-UES (Tables S7 and S8), and JOACMEQ-UEF (Tables S9 and S10) did not overlap with clusters showing a significant FC difference between CM and HC.

### Postoperative changes of FCs in the CM group

**Figure 2:**
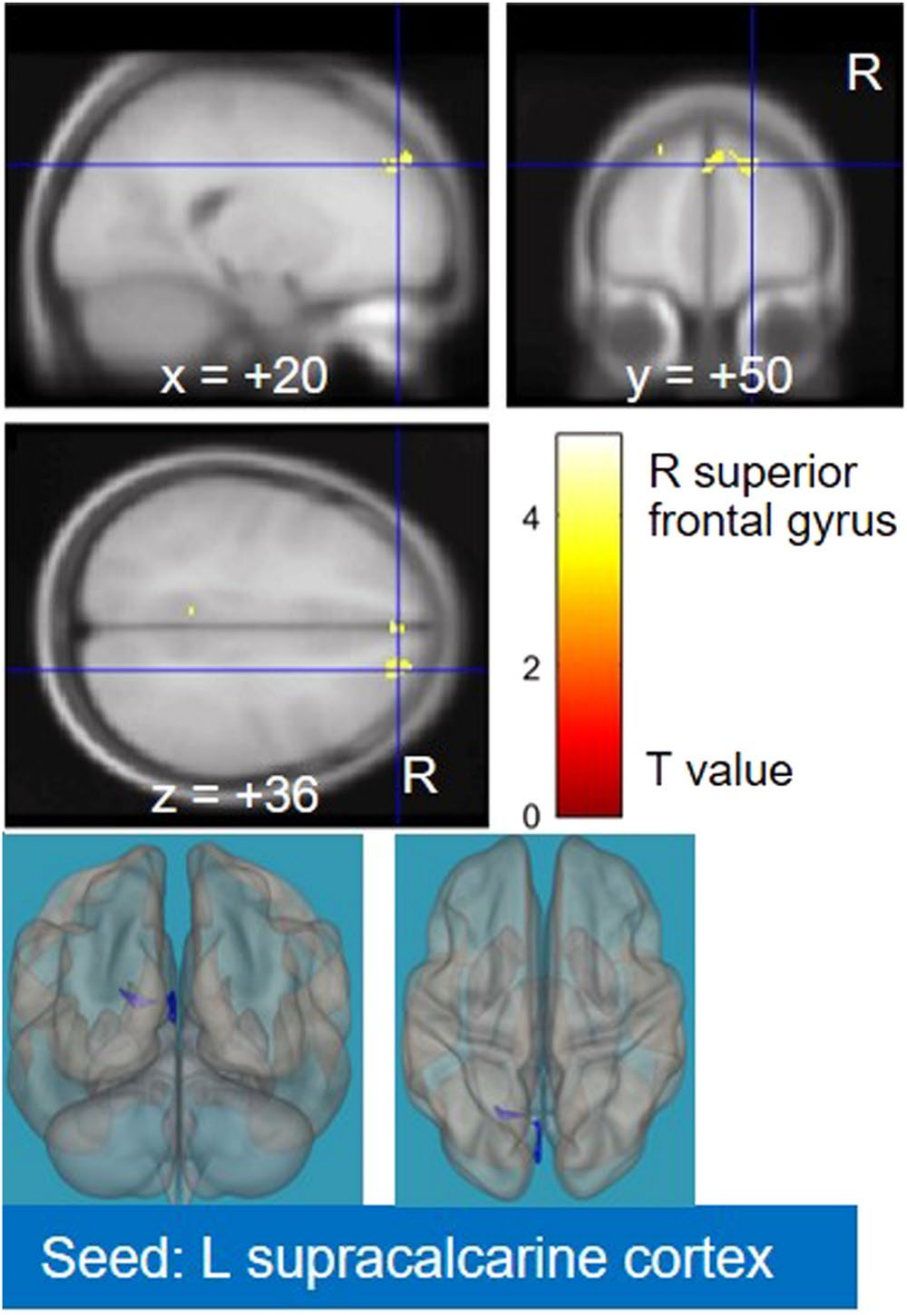
Result of the comparison between the pre- and postoperative cervical myelopathy (CM) groups. After using the CM < healthy control (HC) mask, the left supracalcarine cortex showed decreased connectivity with the right superior frontal gyrus. R, right; L, left.

Comparison of pre- and postoperative CM revealed no cluster showing significantly decreased FC in the CM group (Table S11). In contrast, among 13 clusters showing a significant postoperative increase in the CM group (Table S12), one cluster (an FC of the left supracalcarine cortex with the right superior frontal gyrus) overlapped with clusters showing a significant FC decrease in preoperative CM versus HC (Table S1) (Fig. 2). There was no overlap with clusters showing a significant FC increase in preoperative CM compared with HC.

### Prediction model for the postoperative neurological recovery in CM patients

Our analyses indicated that FCs between the visual cortex and the right superior frontal gyrus showed significant CM-HC differences, significant correlations with postoperative gain in clinical scores (the 10-second test), and significant pre-post differences. We used the conjunction results of the first two analyses to suggest a potential predictive model for clinical recovery in the 10-second test by using FCs of the primary visual cortex, left intracalcarine cortex, and left lingual gyrus with the right superior frontal gyrus as an independent variable (bearing in mind that such prediction models are identified based on the fact that the regions identified come from the same brain-wide search for the factor of interest^23^). Independent variables also included sex, age, and disease duration. However, these 3 demographic variables were removed after forward stepwise selection.

**Figure 3:**
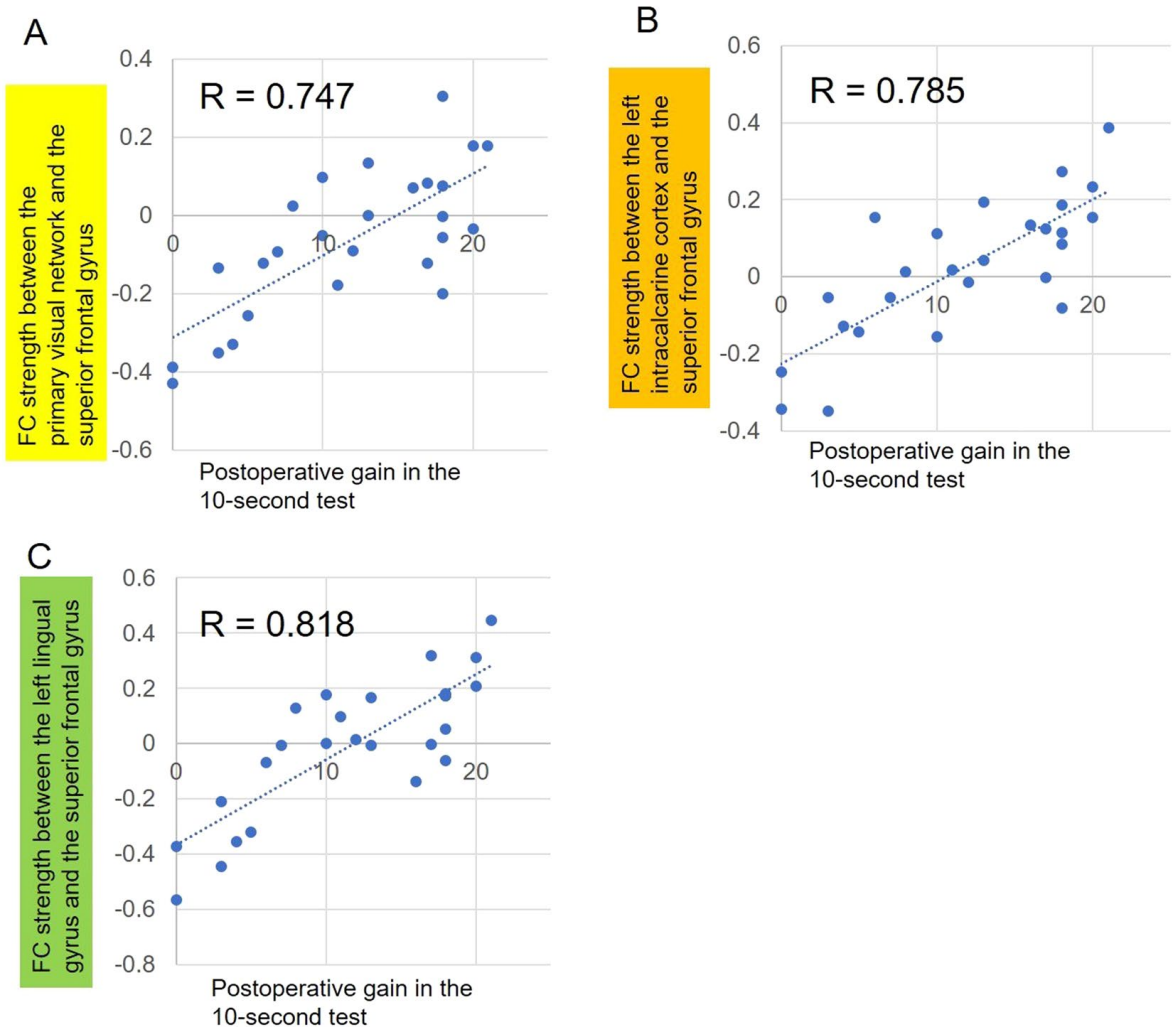
Correlation between postoperative gain in the 10-second test and functional connectivity strength in three visual areas (the primary visual cortex, left intracalcarine cortex, and left lingual gyrus) and the right superior frontal gyrus in patients with cervical myelopathy.

Figure 3 shows the relationship between postoperative gain in the 10-second test and FC strength. We did not apply multiple regression because there was multicollinearity (correlation coefficients more than 0.66) among the three clusters. From the three clusters, we chose FC between the left lingual gyrus and right superior frontal gyrus (x = 28, y = 28, z = 40) for the model development because it showed the highest correlation. The prediction formula developed in this process was as follows: the predicted postoperative gain in the 10-second test = 32.4 × FC strength + 11.9 (R^2^ = 0.669, p < 0.001). Internal validation with 6-fold cross validation indicated an average R^2^ of 0.622.

## Discussion

In this study, we conducted multiple analyses of FC in patients with CM, including (i) comparisons with an HC group, (ii) correlations with clinical scores, and (iii) postoperative changes. Furthermore, we suggest a possible predictive model for postoperative neurologic recovery (postoperative gain in the 10-second test) using preoperative FC. Our results demonstrate the potential utility of brain rs-fMRI as a biomarker for CM.

It is important to note in an initial exploratory study such as this, there was no a priori hypothesis about which seeds should be considered in rs-fMRI studies of CM. To avoid seed selection bias, we used all brain regions as seeds in the inter-group difference analysis. Naturally, this introduces a potential risk of false positive error. Therefore, we used the Bonferroni correction. In the subsequent analyses (the correlation analysis with clinical scores and the pre-post comparison), the use of seeds were limited to 26 that showed significant FC differences in preoperative CM versus HC to focus on the association between preoperative FC decrease or increase in CM and postoperative recovery within the same FCs. As a result, only three clusters (FCs of the primary visual cortex, left intracalcarine cortex, and left lingual gyrus with the right superior frontal gyrus) showed significant inter-group differences and correlations with clinical recovery (postoperative gain in the 10-second test). Importantly, one cluster [an FC of the left supracalcarine cortex (a visual area) and the right superior frontal gyrus] also showed a significant difference in the pre-post comparison.

At first glance, the identification of visual cortical connectivity differences may seem surprising given that CM is not a primary visual disorder. However, a recent study that investigated preoperative visual cortex neural activity and FC alterations in CSM patients revealed the differences between the visual cortex and the posterior cingulate gyrus^18^. Although there is a difference in the location of clusters, CM patients may exhibit changes in the FC of the visual cortex with some clusters. Considering the previous report with our results, the visual cortex may be important for neurological recovery in patients with CM.

Among the three preoperative FCs showing significant CM-HC differences and significant correlations with clinical scores, the FC between the left lingual gyrus and right superior frontal gyrus showed the highest correlation with postoperative gain in the 10-second test. The lingual gyrus is associated with self-referential processing, social cognition^24,25^, and complex visual processing, and it is directly linked to visual memory^26^. Because visual memory plays an important role in perceiving spatial location of extremities^27–29^, the lingual gyrus may be related to neurological recovery of patients with CM.

In terms of clinical significance, we do not think that FC analysis using brain rs-fMRI is necessary for all patients with progressive myelopathy as a preoperative routine imaging assessment. However, in cases with a chronic course lasting for several years^30^, it is difficult for surgeons to decide whether surgical treatment is justified. Such an FC analysis may represent a biomarker that has potential to predict postoperative recovery, whereas further prospective studies are needed to establish the reliability of FC as a biomarker. At the very least, this possibility deserves further, targeted investigation.

Among the four clinical scores associated with upper extremities, only the change in the 10-second test was significantly correlated with preoperative FC values after the masking of CM > HC or CM < HC in the CM patients. One explanation is that the 10-second test is a continuous variable with a large range of values (from 15.6 to 27.4 on average) after surgery compared with other outcome measures (JOA-UEM, JOA-UES, and JOACMEQ-UEF), which represent ordinal variables.

Our study had several limitations. First, only clinical scores for upper extremities were used in the analysis because dysfunction in the lower extremities can be affected by other orthopaedical diseases, such as lumbar spinal canal stenosis and osteoarthrosis in the knee or hip. A multicentre study with a large sample size is now being initiated to establish a comprehensive prediction formula including lower extremity scores and other neurological deficits. Second, we did not perform external validation with the predictive model designed in this study using the FC of the lingual cortex with the superior frontal gyrus in other patient populations. Although this model was internally validated with a 6-fold cross-validation, further prospective studies are needed.

In conclusion, we demonstrated the feasibility of prognostic prediction in terms of the 10-second test using the FC between the visual cortex and the superior frontal gyrus with a three-step analysis for patients with: (i) comparison with HC; (ii) examining correlations with clinical scores; and (iii) analysis of postoperative changes. The current study can, therefore, be considered as providing a framework for designing novel tools to predict the extent of neurological recovery in CM based on brain rs-fMRI.

## Methods

All protocols for this study were approved by Ethical Review Board of Osaka University Hospital (No. 14352-7). The study was performed in accordance with approved guidelines and in compliance with the principles of the Declaration of Helsinki. All participants provided written informed consent before undergoing any study procedure.

### Participants

We recruited 28 patients with CM (male, n = 14; female, n = 14; mean age = 66.5 years, range = 44–79) at our institution from August 2015 to June 2017. Inclusion criteria were: (1) younger than 80; (2) volunteered to enrol in the study; (3) an indication for cervical decompression surgery for cord compression on a cervical spine MRI, such as cervical spondylosis, or ossification of the posterior longitudinal ligament; and (4) existence of myelopathy hand symptoms^19^. Exclusion criteria were: (1) 80 years of age or older; (2) refusal to enrol; (3) traumatic cord compression; and (4) a history of brain diseases such as cerebrovascular lesion or tumour. We selected 28 age- and sex-matched HCs (male, n = 14; female, n = 14; mean age = 66.5 years, range: 43–79) from the HC database in our institution. Detailed demographics are shown in Table 1. We used SPSS statistical software (v. 21.0, IBM Corp., Armonk, NY) for comparisons of demographic data between groups using two-sample t-tests and between pre- and postoperative CM using paired t-tests. The significance level was set at p < 0.05.

Clinical outcomes in the CM group were assessed using scores for the 10-second test^19^, JOA score^20^, JOACMEQ^21^, and visual analogue scale. A previous study demonstrated good correlation of the total score with the grade of myelopathy hand, as assessed by the 10-second test^19^. Among the clinical scores, four upper-extremity scores (10-second test, JOA-UEM, JOA-UES, and JOACMEQ-UEF) were used for the correlation analysis with FCs to avoid the confounding factors such as lumbar degenerative diseases and osteoarthrosis in the lower extremities. Patients underwent an rs-fMRI scans two days before surgery (cervical laminoplasty [N = 25] or anterior decompression and fusion [N = 3]). Subsequently, twenty-six of these patients also underwent an additional rs-fMRI scan six months after surgery.

### Resting state fMRI data acquisition and pre-processing

MRI scans were performed with a 3.0 Tesla MRI scanner (GE, Discovery MR750, Milwaukee, USA). Participants were instructed to simply rest with their eyes closed, not to think of anything in particular, and not to fall asleep. We confirmed the participants’ alertness by checking that they could follow the instructions to raise their hand immediately before and after the resting state scanning. We also confirmed that the participants did not fall asleep during the resting scan by the direct question. Functional images were acquired with a five-minute scan using a blood oxygen level dependent gradient echo-planar pulse sequence (repetition time [TR] = 2000 ms, echo time [TE] = 30 ms, slice thickness = 3.5 mm, in-plane resolution = 3.5 mm × 3.5 mm and flip angle = 90°). Forty axial slices were acquired. Data pre-processing was performed with SPM12 (http://www.fil.ion.ucl.ac.uk/spm/, Wellcome Department of Cognitive Neurology, University College London, London)^31^ and the CONN-fMRI toolbox^32^. For each participant’s data, the first five volumes were discarded, and the following steps were performed: slice timing correction, realignment, co-registration to the participant’s structural image, normalization to the Montreal Neurological Institute (MNI) stereotactic template with a resolution of 2 × 2 × 2 mm cubic voxels, and smoothing with 6-mm full width at half maximum Gaussian kernel. A voxel is a volume element (volumetric and pixel) representing a value in the three-dimensional space, corresponding to a pixel for a given slice thickness. To remove artifacts from non-neural sources, components related to fMRI signals in white matter/cerebrospinal fluid extracted with the CompCor procedure^33^ and six motion parameters were regressed out of the data. Lastly, a bandpass filter (0.008–0.09 Hz) was applied^34,35^.

### Functional connectivity analysis

Functional connectivity analysis was performed with the CONN-fMRI toolbox^32^ for SPM12. We conducted a seed-to-voxel connectivity analysis, which computes correlations of fMRI signal time series between seeds and other voxels of the brain^36,37^. Because of the lack of consensus regarding potential seeds for prediction of preoperative recovery in CM, anatomical regions across the whole brain were used as seeds to avoid seed-selection bias. Specifically, we used 132 anatomical regions based on the Harvard-Oxford atlas and 32 parts of the 8 canonical resting-state networks (default mode, sensorimotor, visual, salience, dorsal attention, frontoparietal, language, and cerebellar network) as seeds for FC analysis. These seed templates were provided by the CONN toolbox. Using 164 seeds leads to false positive error. We used the Bonferroni correction as a rigorous multiple comparison correction method after performing FDR correction for each seed. In the correlation analysis between preoperative FCs and clinical scores, we used only 26 seeds that showed significant FC differences in preoperative CM versus HC (Tables S1 and S2). This is because we hypothesized that preoperative FC differences in CM could be related to postoperative clinical recovery within the same FCs. Therefore, we focused on FC by imposing the condition that an overlap should be observed between correlations with clinical scores and inter-group differences. In the postoperative FC changes in CM, we also used the seeds showing significant FC differences in preoperative CM versus HC. We subsequently focused on the overlap between postoperative changes and inter-group differences based on the hypothesis that preoperative FC differences in CM could be related to postoperative FC normalization within the same FCs.

### Comparisons between preoperative CM and HC and correlation between preoperative FCs and clinical score changes

We performed two-sample t-tests to evaluate inter-group differences between CM patients and HC. The significance levels were set at p < 0.001 (uncorrected, voxel level) and p < 0.000305 (0.05/164; FDR corrected, cluster level and multiple comparison correction [Bonferroni correction]). We also performed correlation analyses to identify relationships between preoperative FCs strength and postoperative gain in clinical scores (the 10-second test, JOA-UEM, JOA-UES, and JOACMEQ-UEF). In addition to the inter-group difference analysis, whole-brain regression analysis was employed to detect relationships between preoperative FCs and clinical score changes in the CM group.

We extracted the connectivity coefficients between the seeds and the target regions, which were then correlated with clinical scores using Spearman’s rank correlation coefficient. Firstly, we performed correlation analysis on CONN for each preoperative FC with significance thresholds of p < 0.001 (uncorrected, voxel level), and p < 0.00192 (0.05/26; FDR corrected, cluster level and Bonferroni correction). We then checked for overlap between clusters showing a significant correlation with clinical scores and clusters showing a significant CM-HC difference by masking the results of correlation with clinical scores using contrasts obtained from CM-HC differences. In this step, we applied a small volume correction on the masked regions. The significance levels were set at p < 0.001 (uncorrected, voxel level) and p < 0.05 (FDR corrected, cluster level).

### Comparisons between preoperative CM and HC and between pre- and postoperative CM

We also performed paired t-tests to evaluate postoperative changes in CM patients. In these comparisons, the significance levels were set at p < 0.001 (uncorrected, voxel level) and p < 0.00192 (0.05/26; FDR corrected, cluster level, and Bonferroni correction). Subsequently, we checked for overlap between clusters showing a significant pre-post difference and clusters showing a significant CM-HC difference, by masking the results of pre-post differences using contrasts obtained from CM-HC differences. In this step, we applied a small volume correction on the masked regions. The significance levels were set at p < 0.001 (uncorrected, voxel level) and p < 0.05 (FDR corrected, cluster level).

### Development and validation of the prediction model

A prediction formula was provided using the regression model for clinical score changes, in which independent variables were chosen with a forward stepwise selection including a significant preoperative FC, sex, age and the disease duration. Internal validation was conducted with 6-fold cross-validation. The number of data subsets was set based on Sturges’ formula^38^. Six-fold cross-validation involves randomizing the data set into 6 separate and unique train and test sets. Each set consists of a training set comprising 83% of preoperative CM patients and a test set consisting of the remaining 17% of patients. To reduce the arbitrariness in the choice of random number seeds, the results were averaged by repeating the 6-fold cross validation 5 times. A regression model was plotted to evaluate the predictive accuracy and robustness of the models. We used SPSS for calculating Pearson’s correlation coefficient, prediction model generation, and internal validation. The significance level was set at p < 0.05.

## Supplementary information

Supplementary Tables

## Data Availability

The datasets generated during and/or analysed during the current study are available from the corresponding authors on reasonable request.
